# Characterization of Phytoplankton-Derived Amino Acids and Tracing the Source of Organic Carbon Using Stable Isotopes in the Amundsen Sea

**DOI:** 10.3390/md22100476

**Published:** 2024-10-18

**Authors:** Jun-Oh Min, Min-Seob Kim, Boyeon Lee, Jong-Ku Gal, Jinyoung Jung, Tae-Wan Kim, Jisoo Park, Sun-Yong Ha

**Affiliations:** 1 Division of Ocean and Atmosphere Sciences, Korea Polar Research Institute (KOPRI), Incheon 21990, Republic of Korea; jomin8310@gmail.com (J.-O.M.); boyeon@kopri.re.kr (B.L.); jinyoungjung@kopri.re.kr (J.J.); twkim@kopri.re.kr (T.-W.K.); jspark@kopri.re.kr (J.P.); 2 Environment Measurement and Analysis Center, National Institute of Environmental Research (NIER), Incheon 22689, Republic of Korea; candyfrog77@gmail.com; 3 East Sea Fisheries Research Institute, Environment and Fisheries Resources Research Division, National Institute of Fisheries Science (NIFS), Gangneung-si 25435, Republic of Korea; jkgal@gmail.com

**Keywords:** Amundsen Sea, antarctica, amino acid, *P. antarctica*, stable isotope

## Abstract

We utilized amino acid (AA) and carbon stable isotope analyses to characterize phytoplankton-derived organic matter (OM) and trace the sources of organic carbon in the Amundsen Sea. Carbon isotope ratios of particulate organic carbon (δ^13^C-POC) range from −28.7‰ to −23.1‰, indicating that particulate organic matter originated primarily from phytoplankton. The dissolved organic carbon isotope (δ^13^C-DOC) signature (−27.1 to −21.0‰) observed in the sea-ice melting system suggests that meltwater contributes to the DOC supply of the Amundsen Sea together with OM produced by phytoplankton. A negative correlation between the degradation index and δ^13^C-POC indicates that the quality of OM significantly influences isotopic fractionation (r^2^ = 0.59, *p* < 0.001). The AA distribution in the Amundsen Sea (5.43 ± 3.19 µM) was significantly larger than previously reported in the Southern Ocean and was associated with phytoplankton biomass (r^2^ = 0.49, *p* < 0.01). Under conditions dominated by *P. antarctica* (DI = 2.29 ± 2.30), OM exhibited greater lability compared to conditions co-dominated by diatoms and *D. speculum* (DI = 0.04 ± 3.64). These results highlight the important role of *P. antarctica* in influencing the properties of OM, suggesting potential impacts on carbon cycling and microbial metabolic activity in the Amundsen Sea.

## 1. Introduction

Amino acids (AAs) constitute a major portion of the living biomass and nonliving particulate organic matter (POM) and dissolved organic matter (DOM) pools in marine systems [1,2]. The carbon-normalized yield (AA-C %) and degradation index (DI) of AAs are biogeochemical indicators of organic matter (OM) cycling, transformation, and decomposition processes [3,4]. The high bioavailability of particulates, such as the L-enantiomer of AAs (L-AAs) and neutral amino sugars, stimulates microbial activity and leads to rapid remineralization [1]. In addition, during organic decomposition, microorganisms can leave traces on POM biomolecules such as D-enantiomers of AAs (D-AAs) and muramic acids [5]. The abundance of D-AAs directly correlates with both bacterial growth and degradation of OM [6] and has been widely used to estimate the contributions of bacterial-derived organic carbon (OC) and nitrogen in sediments, particles, and the DOM pool [5,7,8].

Since POM and DOM are derived from various sources, the carbon stable isotope ratio (δ^13^C) and C/N ratio are used as proxies for tracing the source of marine OM [9,10]. Marine environments exhibit heavy δ^13^C values (1‰) due to newly photosynthesized OM formation, whereas terrestrial environments have lighter δ^13^C values (−8‰ to −9‰) due to atmospheric CO_2_ fixation [9]. The fractionation differences between marine and terrestrial environments result in marine planktonic OM with relatively enriched dissolved organic carbon isotope (δ^13^C-DOC) signatures, ranging from −19‰ to −24‰. In contrast, typical terrestrial OM shows a δ^13^C-DOC signature of approximately −28‰ [9,11]. The natural variation in δ^13^C-DOC is useful for observing the salinity continuum in freshwater systems, where δ^13^C-DOC signatures are sufficiently distinct to differentiate between freshwater and marine OM [12]. The C/N ratio of terrestrial origin OM is generally greater than 12, while the phytoplankton C/N ratio is between 6 and 8, differentiating allochthonous from autochthonous sources [13]. In Antarctica, the majority of DOC is derived from phytoplankton [14,15], with a significant portion also contributed by meltwater [16,17,18]. Therefore, δ^13^C-DOC signatures and C/N ratios are effective tools for tracing the sources and transformations of mixed DOC pools.

The Southern Ocean is an important carbon sink responsible for approximately 20% of the global ocean’s absorption of CO_2_ from the atmosphere [19,20]. The Amundsen Sea Polynya (ASP) is a seasonally recurring area of open water surrounded by sea ice and a “hot pot” for energy and mass transfer between the atmosphere and polar seas [21]. Among the 37 polynyas that form along the coast of the Southern Ocean, the ASP is the most biologically productive region, with annual primary productivity measured based on satellite observations reaching 160 ± 36.9 g C m^−2^ per year [22]. Phytoplankton growth in the ASP is predominantly driven by the haptophyte *Phaeocystis antarctica* and diatoms. *P. antarctica* blooms occur in the surface mixed layer and can persist for much of the austral summer season [22,23]. Previous studies conducted in the Ross Sea have shown that *P. antarctica* accounts for more than 60% of seasonal primary production [24]. Due to its ubiquity and high productivity associated with blooms, *P. antarctica* is an important component of marine POM and DOM [25]. These blooms contribute OC and nutrients to the food web within the ASP, creating an ecological niche for microbial heterotrophs [25,26].

The amount of OC discharged through Antarctic ice-sheet meltwater is estimated to be 0.33 Tg C per year as particulate organic carbon (POC) and 0.17 Tg C per year as DOC [18]. The Amundsen/Bellingshausen sector in West Antarctica has experienced the greatest mass loss, with an annual loss of 159 ± 8 Gt per year [27]. Warm Circumpolar Deep Water (CDW) introduced into the Amundsen Sea continental shelf is accelerating the basal melting of the Getz and Dotson Ice Shelves [28,29]. The cumulative mass losses of the Getz and Dotson Ice Shelves observed between 1979 and 2017 were 363 and 211 Gt, respectively [27]. Glacier meltwater supplies significant amounts of OC, nutrients, and trace nutrients to the water column [17,18], which can stimulate primary production and change the structure of microbial communities [30,31].

Previous studies of the sources and properties of OM in the Amundsen Sea have focused mainly on the macromolecular composition of POM [32,33] and the fluorescence properties of DOM [34,35,36]. Despite the importance of research on the sources and characteristics of OM, detailed qualitative and quantitative analyses of OM using AA composition and δ^13^C data were conducted for the first time in the Amundsen Sea. Therefore, the objectives of this study were (1) to characterize the organic molecules in phytoplankton communities and (2) to understand the sources and transformation processes of OM associated with the biological carbon pump.

## 2. Results

### 2.1. Hydrography

Seawater is divided into three main water masses in the Amundsen Sea. The Antarctic Surface Waters (AASW) form a relatively warm, low-salinity freshwater mass caused by solar heating and meltwater inflow by sea ice melt. Saltier and colder Winter Waters (WW) from sea ice formation in the previous winter and modified Circumpolar Deep Water (mCDW) with warmer and saltier characteristics are observed in the bottom layer [37,38].

**Figure 1:**
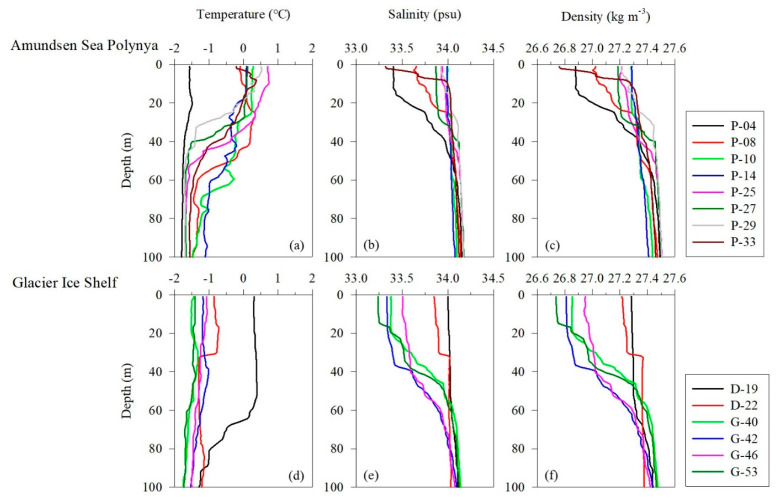
Vertical profiles of physical parameters in the Amundsen Sea. (**a**–**c**) Temperature, salinity, and density in the Amundsen Sea Polynya. (**d**–**f**) Temperature, salinity, and density in the glacier ice shelf.

The vertical distributions of water temperature, salinity, and density in this study area ranged from −1.80 to 0.74 °C (mean, −1.03 ± 0.16 °C), 33.2 to 34.2 psu (mean, 33.9 ± 0.13 psu), and 26.7 to 27.5 kg m^−3^ (mean, 27.3 ± 0.10), respectively (Figure 1). The average temperature pooled from the surface and subsurface chlorophyll maximum layers (SCMs) was −0.37 ± 0.82 °C (range, −1.57–0.73 °C), the salinity was 33.8 ± 0.26 psu (range, 33.2–34.0 psu), and the density was 27.1 ± 0.18 kg m^−3^ (range, 26.7–27.3 kg m^−3^) (Table 1). The physical parameters at the depth where the samples were collected were well defined for the AASW (S = <34.1 psu, T = −1.80 to >0 °C) water mass feature [38]. Pycnocline formation was observed at sites P-4, P-8, P-27, and P-33 in the Amundsen Sea Polynya (ASP) region and at locations D-22, G-40, G-42, and G-53 in the ice shelf region (Figure 1c,f). Satellite images show that sea ice is widely distributed (>90%) around the ice shelf margin and the surface around the ASP. These results suggest that low-salinity meltwater derived from the sea ice contributed to the formation of pycnocline (Figure 1b,c,e,f). The average mixed layer depths (MLD) at the Dotson Ice Shelf (DIS) and Western Getz Ice Shelf (WGIS) were 48 ± 25 m and 32 ± 17 m, respectively (Table 1). In particular, the formation of an MLD approximately 2.5 times deeper than average was observed at the D-19 station (65 m) in the DIS front (Table 1). These results suggest that mCDW inflow along the Dotson Trough destabilizes the water column due to the upwelling of mCDW mixed with buoyant basal glacial meltwater from the DIS, thereby increasing the MLD [39].

### 2.2. Phytoplankton Biomass and Structure

The chlorophyll-a (Chl-a) concentrations measured on the surface and in the SCMs layer in the ASP ranged from 1.57 to 11.9 µg L^−1^ (Table 1). A high Chl-a concentration (11.9 µg L^−1^) was detected in the SCMs of the P-25 region of the ASP. The Chl-a concentrations measured in the ASP (mean, 4.60 ± 3.25 µg L^−1^) were approximately two-fold lower than those reported from December 2013 to January 2014 (mean, 8.89 ± 1.11 µg L^−1^) [40]. Phytoplankton blooms began in the northeastern part of the ASP during the study period, and at the time of sampling, the ASP site was not yet fully affected by the blooms [41]. The Chl-a concentrations in the DIS and WGIS ranged from 2.51 to 5.26 μg L^−1^ (mean, 3.71 ± 1.18 μg L^−1^) and from 0.70 to 6.15 μg L^−1^ (mean, 4.04 ± 1.57 μg L^−1^), respectively.

The major phytoplankton groups in the ASP observed by microscopic identification include *P. antarctica*, diatoms, and *D. speculum* [42]. Lee et al. [42] reported that the microscopic phytoplankton biomass in the central polynya was dominated by large pennate diatoms (31%), centric diatoms (14%), and *D. speculum* (48%) rather than *P. antarctica* (3%).The microscopic results were similar to the phytoplankton community structure determined using the disappearance nutrient ratio (NO_3_:PO_4_) as a chemical proxy (Figure S1). The NO_3_:PO_4_ ratio in the center of the ASP (slope = 13) was higher than that observed under diatom bloom conditions reported in previous studies (slope = 10) [43], indicating that the co-dominance of diatoms and *D. speculum* contributed to the NO_3_:ΔPO_4_ ratio. The NO_3_:ΔPO_4_ ratio (slope = 17) in the eastern ASP track (P-25, 27, 29) and at station P-04 was similar to the *P. antarctica* ratio observed in the Amundsen Sea [44]. Indeed, microscopic identification revealed that *P. antarctica* accounted for 92% of the abundance at station P-04 [42]. The combined NO_3_:PO_4_ ratios (slope = 15) from the DIS and WGIS exhibited intermediate values between those of the ASP center and the eastern sectors, suggesting a mixed phytoplankton structure.

### 2.3. Chemical Parameters

The concentration ranges of NO_3_ and PO_4_ at the sample collection depth in the ASP were 5.60 to 22.4 µM (mean, 15.5 ± 4.91 µM) and 0.65 to 1.54 µM (mean, 1.18 ± 0.30 µM), respectively (Table 1). In the DIS, the average NO_3_ concentration was 20.4 ± 3.22 µM (range, 17.3–23.7 µM), and the average PO_4_ concentration was 1.65 ± 0.29 µM (range, 1.38–1.92 µM). NO_3_ was at slightly lower concentrations (mean, 14.5 ± 2.55 µM; range, 11.8–19.2 µM) in the WGIS than at the other study sites, and PO_4_ (1.22 ± 0.24 µM) was at similar concentrations.

The particulate organic carbon (POC) concentrations were slightly higher in the ASP (mean, 67 ± 16 µM; range, 48–117 µM) than in the DIS (mean, 62 ± 14 µM; range, 45–76 µM) and WGIS (mean, 65 ± 7 µM; range, 52–71 µM) (Table 2). The concentration of particulate organic nitrogen (PON) (mean, 9 ± 2 µM; range, 6–15 µM) was slightly higher in the ASP than in the DIS (mean, 7 ± 4 µM; range, 3–11 µM) and WGIS (mean, 7 ± 3 µM; range, 3–11 µM), similar to the distribution of POC concentrations (Table 2). The highest mean POC and PON concentrations at the P-25 site were associated with high Chl-a (9.8–11.9 µg L^−1^). The average POC/PON mean for this study area was found to be 9 ± 1 and was within the ratios (range, 5.4–9.2) previously reported for the Ross Sea [45].

The average concentration of dissolved organic carbon (DOC) measured in this study ranged from 44 to 45 µM and was distributed similarly to that in most of the study areas (Table 2). The mean concentrations of dissolved organic nitrogen (DON) in the ASP, DIS, and WGIS were 2 ± 1 µM (range, 1–4 µM), 3 ± 1 µM (range, 2–4 µM), and 3 ± 2 µM (range, 1–5 µM), respectively. The concentrations of DOC and DON observed in this study were lower than those previously reported during the early to mid-phytoplankton bloom in the Amundsen Sea (DOC, 53–127 µM; DON, 0–8.5 µM) [46]. DOC/DON ratios were higher in the ASP (mean, 27 ± 14; range, 11–58) than in the WGIS (mean, 22 ± 15; range, 8–63), with the lowest ratios (mean, 17 ± 4; range, 13–21) in the DIS.

In this study, particulate organic carbon isotope (δ^13^C-POC) was observed in the range of −28.7 to −23.1‰ (Table 2). The mean δ^13^C-POC values for the ASP, DIS, and WGIS were −25.8 ± 1.26‰, −27.3 ± 1.05‰, and −24.5 ± 1.12‰, respectively. The δ^13^C-POC in the surface layer at site D-22 was of relatively light value (−28.7‰), and the heaviest isotope ratio (−23.1 ‰) was detected in the SCMs at sites G-40 and G-53. The dissolved organic carbon isotope (δ^13^C-DOC) signature in the study area ranged from −33.3 to −21.0‰ (Table 2). Light δ ^13^C-DOC was observed at the P-4 site (−33.3‰), and the heaviest δ^13^C-DOC was observed at the P-29 site (−21.0‰) and in the SCMs of D-19 (−21.0‰).

### 2.4. Amino Acid Composition in Particulate Organic Matter

#### 2.4.1. Amino Acid Composition

The particulate amino acid (PAA) concentrations in the ASP ranged from 0.83 to 7.00 µM (mean, 3.01 ± 1.81 µM) (Table 2). The high PAA concentrations at the P-25 site (mean, 7.00 ± 0.01 µM) in the ASP were consistent with the high POC, PON, and Chl-a concentrations. The PAA concentrations in the DIS ranged from 8.24 to 10.2 µM (mean, 9.05 ± 0.92 µM), with the highest PAA concentration (10.2 μM) found in the surface layer at site D-22. The average PAA concentration was 4.24 ± 2.63 μM (range, 1.62–8.33 µM) in the WGIS (Table 2).

**Figure 2:**
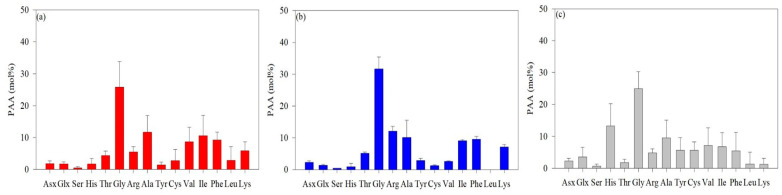
The average concentrations of L-enantiomer amino acids in particulate organic matter in the Amundsen Sea Polynya (ASP) (**a**), the Dotson Ice Shelf (DIS) (**b**), and the Western Getz Ice Shelf (WGIS) (**c**). Fourteen standard solutions were used in L-AA analysis: asparagine + aspartic acid (Asx), glutamine + glutamic acid (Glx), serine (Ser), histidine (His), glycine (Gly), threonine (Thr), arginine (Arg), alanine (Ala), tyrosine (Tyr), valine (Val), phenylalanine (Phe), isoleucine (Ile), leucine (Leu), and lysine (Lys).

The average AA concentrations measured in the surface layer and SCMs are shown in Figure 2. The predominant AAs identified in the ASP were Gly, Ala, Val, Ile, and Phe. The average concentration of Gly was 25.8 mol%, which was significantly higher than that of the other AAs, with Ala having the second-highest average concentration at 11.7 mol% (Figure 2a). The average molar concentrations of Ile, Phe, and Val were 10.6 mol%, 9.27 mol%, and 8.73 mol%, respectively (Figure 2a). The concentration of Gly in the DIS was 31.7 mol%, the highest in the Amundsen Sea, with Arg and Ala concentrations at 12.1 mol% and 10.3 mol%, respectively. (Figure 2b). The Gly concentrations in the WGIS (24.9 mol%) were lower than those in the ASP and DIS (Figure 2c). The average concentration of His was 13.3 mol%, which was much greater than that of the ASP (1.68 mol%) and DIS (0.91 mol%).

#### 2.4.2. Indicators of Particulate Organic Matter Diagenesis

The particulate amino acid carbon yield (PAA-C), the percentage of D-enantiomers amino acid (D-AA%), and the bacterial-C yield were used to assess diagenetic changes in particulate organic matter (POM), while a degradation index (DI) was calculated to evaluate the state of POM. (Table 2). The PAA-C in POM varied between 5.65 and 82.7% depending on the study area. In the ASP, the PAA-C range and mean values were 5.65–45.3% and 20.6 ± 10.4%, respectively. The average PAA-C was 69.5 ± 15.2% at the DIS and 33.3 ± 24.8% at the WGIS. High PAA-C (82.7%) was observed at the SCMs at site D-22.

To estimate the bacterial contribution to POC, the bacterial-C yield was calculated using D-Glx as a proxy (Table 2). The average bacterial-C yields were high in the ice shelf areas, including DIS (mean, 16.3 ± 4.57%; range, 12.0–22.6%) and WGIS (mean, 15.3 ± 9.48%; range, 3.16–26.0%). Notably, the contribution of bacterial-C yield (26%) to POC was highest in the surface layer of station G-40. These results suggest that the bacterial contribution to POM in WGIS is substantial. In comparison, the average bacterial-C yield in the ASP region (mean, 6.75 ± 4.65%; range, 1.48–15.4%) was approximately two-fold lower than in DIS and WGIS (Table 2).

The DI has been used to determine the degradation state of OM, with fresh OM typically yielding positive DI values, while degraded OM yields negative values [4]. In this study, the AA molar ratios of 15 samples were used in principal component analysis (PCA) to derive principal components characterized by maximum variation along the first axis, with the score on that axis considered an indicator of the degradation state of OM (Table 3).

The first axis of the PCA explained 25% of the total variation, and the second axis explained 19% of the total variation (Table 3). The DI calculated in this study ranged from −5.09 to 5.00 and varied significantly among the study regions (Table 2). The mean DI value of the DIS (4.66 ± 0.28) was greater than that of the ASP (1.14 ± 3.17) and WGIS (−4.62 ± 0.66), indicating that the POM composition of the DIS is fresh OM. In contrast, the DI values for most sites on the WGIS were negative, indicating enhanced degradation of POM.

## 3. Discussion

### 3.1. Sources and Stable Isotope Characteristics of Particulate Organic Matter

**Figure 3:**
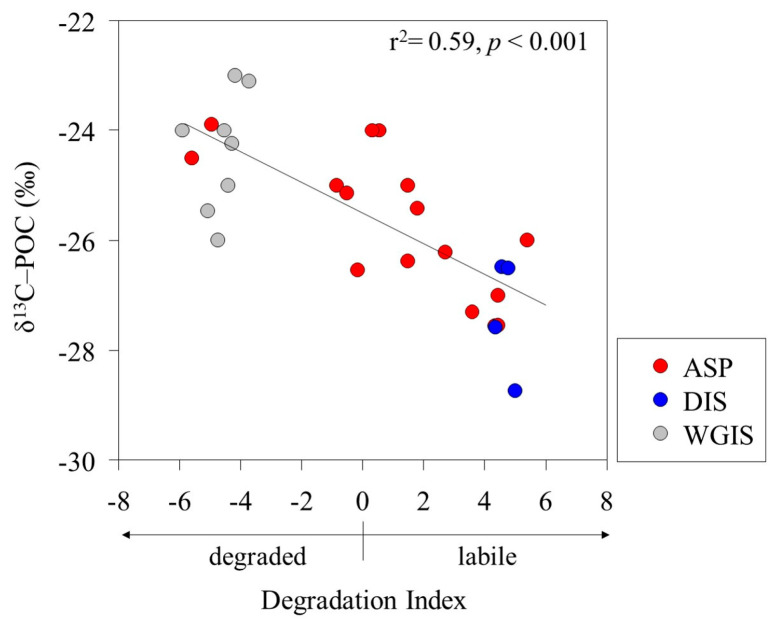
Relationship between the degradation index (DI) and carbon stable isotopes (δ^13^C-POC) of particulate organic matter.

The POC/PON ratio and δ^13^C signature were used as proxies to determine the POM source in the Amundsen Sea. The POC/PON ratio of phytoplankton-derived POM previously studied in the Amundsen Sea was observed to be between 6.3 and 9.2, with the δ^13^C-POC signature observed to be between −28.6 ‰ and −25.4 ‰ [33]. The POC/PON ratio (mean, 9 ± 1) and δ^13^C-POC signature (mean, −25.9 ± 1.4 ‰) results observed in this study showed that most POM was composed of phytoplankton (Table 2). The fractionation of δ^13^C-POC has different δ^13^C values due to various organic sources and biosynthetic pathways [47,48]. For example, polysaccharides and proteins generally have higher δ^13^C values than lipids [49]. Additionally, selective degradation of labile components can significantly alter the δ^13^C composition of POM [50,51]. Interestingly, a significant negative correlation was found between the DI and δ^13^C-POC signatures (r^2^ = 0.59; *p* < 0.001), suggesting that δ^13^C-POC fractionation depends on the state of the POM (Figure 3). Organic compounds primarily composed of labile AAs and neutral sugars are enriched in δ^13^C [52,53]. In contrast, our results show that labile POM, characterized by a positive DI, has a lower δ^13^C-POC compared to degraded POM with a negative DI.

Guo et al. [53] reported that adding fresh plankton-derived OM rapidly stimulated the activity of heterotrophic bacteria and removed a portion of labile δ^13^C-enriched POM by bacterial respiration, thereby reducing bulk δ^13^C-POC values. Considering the high bacterial respiration in the water column in our previous study in the ASP [15], this implies that the stimulation of microbial decomposition activity influences δ^13^C-POC fractionation.

### 3.2. Amino Acid Composition by Phytoplankton Community

In the present study, three distinct groups were identified based on the phytoplankton community structure to determine the diagenetic status of the POM. Group 1 is characterized by the co-dominance of diatoms and *D. speculum* (NO_3_:PO_4_ = 13.0), Group 2 is dominated by *P. Antarctica* (NO_3_:PO_4_ = 17), and Group 3 shows a mixed phytoplankton structure in the glacial ice shelf, including DIS and WGIS (NO_3_:PO_4_ = 15) (Figure S1).

**Figure 4:**
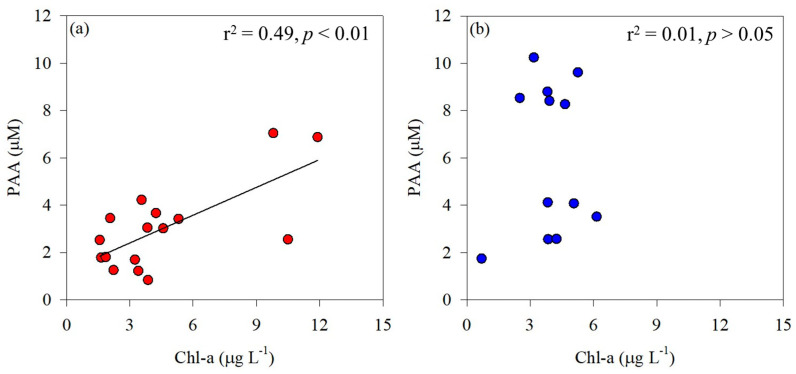
Correlation between phytoplankton biomass (Chl-a) and particulate amino acids (PAA) in the Amundsen Sea Polynya (**a**) and the glacier ice shelf (**b**).

The PAA concentrations measured in the Amundsen Sea ranged widely (0.83–10.2 μM; Table 2) and were much greater than the values previously reported in the Southern Ocean. For example, the PAA concentrations observed in the Ross Sea ranged from 0.18 to 1.04 μM [54], and the PAA concentrations measured in the Weddell Sea ranged from 0.75 to 0.60 μM [55]. Tremblay et al. [56] reported the AA composition and concentrations in POM associated with phytoplankton blooms in a study conducted on Kerguelen Island in the Southern Ocean. In the present study, a significant correlation was observed between phytoplankton biomass and PAA in the ASP (r^2^ = 0.49, *p* < 0.01; Figure 4a), indicating that the distribution of total hydrolyzable PAA is significantly influenced by phytoplankton biomass. According to Jo et al. [54], Chl-a concentration in the Ross Sea ranged from 0.3 to 1.5 µg L^−1^. In contrast, the concentrations observed in this study were considerably higher, ranging from 1.57 to 11.9 µg L^−1^. This suggests that the relatively high PAA concentrations observed in the Amundsen Sea are closely associated with phytoplankton biomass. Unlike in the ASP, there was no significant correlation between phytoplankton biomass and PAA concentrations in the glacial ice shelf, including DIS and WGIS (*p* > 0.05; Figure 4b), implying that the AAs originate from various detritus and meltwater.

**Figure 5:**
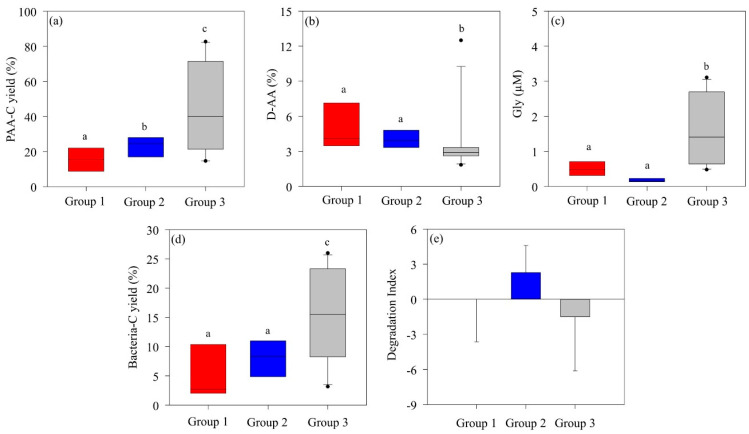
Differences in the (**a**) amino acid carbon yield (PAA-C yield %), (**b**) D-enantiomeric amino acid proportion (D-AA %), (**c**) glycine (Gly) concentration, (**d**) bacterial carbon yield (bacterial-C yield), and (**e**) degradation index of particulate organic matter in each group in the Amundsen Sea. The solid line inside the box represents the median. Boxes represent the 25th to 75th percentiles, and range bars represent the 5th to 95th percentiles. Analysis was performed using the nonparametric Kruskal–Wallis test (*p* < 0.05) followed by the Mann–Whitney post hoc test. Medians following the same letter are not significantly different, as determined by a Mann–Whitney post hoc test (*p* < 0.05).

The AA composition of highly bioavailable OM typically exhibits abundant PAA-C yields while containing relatively low levels of Gly, D-AAs, and nonprotein AAs (β-alanine and γ-aminobutyric acid) [57]. In Group 2, dominated by *P. antarctica*, the PAA-C (mean, 24.5 ± 10.5%) was significantly higher than in Group 1 (mean, 16.6 ± 9.33%), where diatoms and *D. speculum* co-dominated. On the other hand, there were no significant differences in D-AA or Gly concentrations (Figure 5a–c). However, the DI values of Group 2 (mean, 2.29 ± 2.30) indicate a much more labile POM state compared to the DI values of Group 1 (mean, 0.04 ± 3.64) (Figure 5e). During *P. antarctica* blooms, the formation of slime colonies produces bioavailable, labile OM, suggesting that the structured organic particles within *P. antarctica* communities consist of more labile OM than those in communities co-dominated by diatoms and *D. speculum* [25]. Group 3, characterized by mixed phytoplankton structure, showed low DI (mean, −1.52 ± 4.56) and high Gly concentrations, suggesting an enhanced degraded state of POM. In particular, the high bacterial-C yield (15.6 ± 4.60%) indicated that POM was further degraded through bacterial decomposition activity and converted to an altered OM state. In addition, the high PAA-C yield indicated that the contribution was from more refractory OM rather than fresh and labile phytoplankton-derived OM.

### 3.3. Tracing of DOM Sources Using Carbon Stable Isotopes

**Figure 6:**
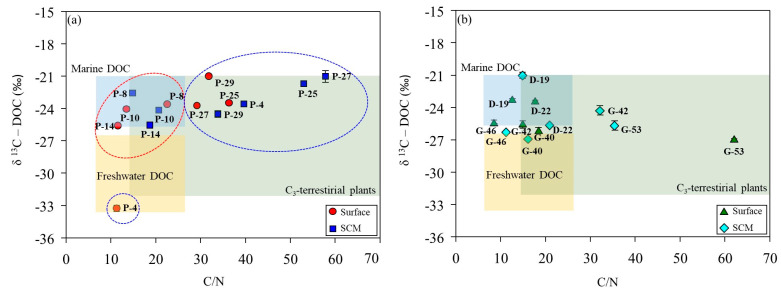
Relationship between the DOC/DON ratio and carbon stable isotope value (δ^13^C-DOC) in the Amundsen Sea Polynya (**a**) and glacial ice shelf areas (**b**). The ranges of DOC/DON ratios and δ^13^C-DOC values for each study area were based on values reported by Lamb et al. [10]. The red circles indicate conditions where diatoms and *Dictyocha speculum* are co-dominant, while the blue circles indicate conditions where *P. antarctica* is dominant.

**Figure 7:**
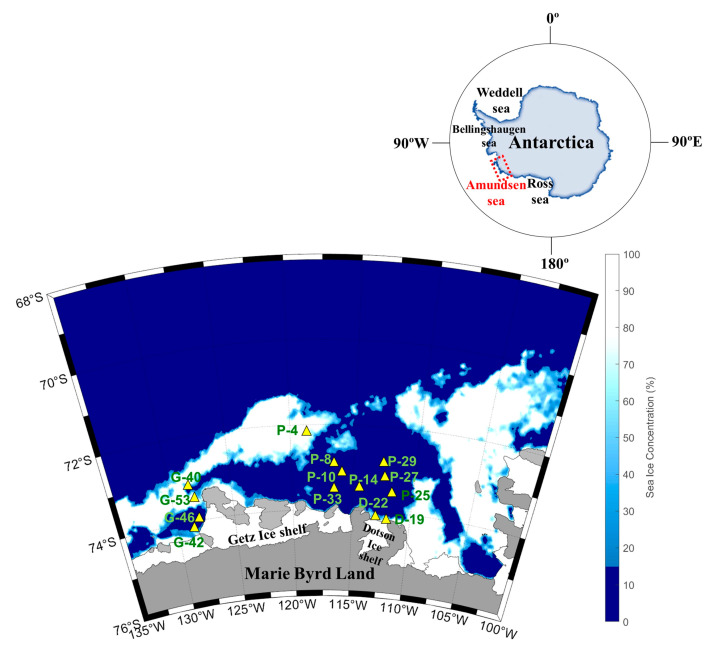
Map of the sampling locations and sea ice distribution in the Amundsen Sea during the austral summer of 2016.

In the ASP, the δ^13^C-DOC signatures at the stations co-dominated by diatoms and *D. speculum* ranged from −24.2‰ to −22.6‰ (mean, −23.6‰), which is characteristic of marine-derived DOM (Table 2; Figure 6a). The surface layer at P-4 displays low salinity (33.4 psu) and depleted δ^13^C-DOC signatures (−33.3‰), indicating a freshwater origin associated with meltwater input from surrounding sea ice (Figure 7). The δ^13^C-DOC signature and DOC/DON ratio increased under conditions dominated by *P. antarctica*. This increase in the DOC/DON ratio (36.8 ± 15.5) suggests that the production of bioavailable labile OM, rich in carbohydrates and mucopolysaccharides, by mucus colonies during the blooming period of *P. antarctica* is reflected in the δ^13^C-DOC signature [25]. The δ^13^C-DOC signature of the DIS is characteristic of marine origin (range, −25.7 to −21.0‰; mean, −23.4 ± 1.89‰), and the SCMs at station D-22 (−25.7‰) fall within a mixed line of marine and freshwater origin (Figure 6b). Fang and Kim [16] reported that DOC concentrations in bottom waters near the DIS were positively correlated with salinity, indicating an additional carbon source associated with the freshwater influx observed in the bottom waters near the DIS. In this study, the physical parameters of DIS suggest the upwelling of mCDW mixed with basal meltwater (see, Section 2.1), supporting the addition of DOC derived from basal meltwater to the water column.

In WGIS, the δ^13^C-DOC signature represents an intermediate value between marine and freshwater sources (Figure 6b). The WGIS region is characterized by extensive sea ice coverage (>50%, Figure 7) and relatively low surface salinity (range, 33.2–33.6 psu). Shallow bottom ice depths in the WGIS prevent the influx of warm mCDW, preventing the addition of basal meltwater to the water column [58]. This suggests that DOC derived from surface meltwater was mixed with DOC derived from phytoplankton and reflected in δ^13^C-DOC values. High DOC/DON at G-42 and G-53 in the WGIS is associated with a DOM pool supplied by the surrounding sea ice (Figure 6b). High DOC/DON ratios in sea ice melting systems were previously reported in Antarctic and Arctic sea ice and explained by the uncoupling of C and N metabolism [59,60]. Thomas et al. [60] reported that the DOM pool in sea ice exhibits elevated DOC/DON ratios, driven by the rapid hydrolysis and microbial utilization of N-rich amino acids compared to C-rich polysaccharides. Therefore, the δ^13^C-DOC signatures and elevated DOC/DON ratios observed in the meltwater system suggest that meltwater contributes to the DOC supply in the Amundsen Sea, together with OM produced by phytoplankton.

### 3.4. Influence of Phytoplankton Community Shifts on the Biological Carbon Pump

Rapid climate change could significantly alter the structure and function of phytoplankton communities in the Southern Ocean, potentially impacting the efficiency of the biological carbon pump [61,62]. Colonial *P. antarctica* plays a major role in the Southern Ocean’s carbon cycle, contributing 46% of the annual net production at 60°S and accounting for 40% of POC exports [43,63]. In this study, the DI values and δ^13^C-DOC signatures suggest that *P. antarctica*-dominated conditions produced significantly more labile OC than those co-dominated by diatoms and *D. speculum* (Figure 5e and Figure 6a). Additionally, the enriched δ^13^C-DOC signature and high DOC/DON ratio further indicate the presence of labile OM under *P. antarctica*-dominated conditions. Ducklow et al. [64] found that under *P. antarctica* dominance in the ASP, 1.58% of net primary production was transported to the deep sea, with most of the exported OM mineralized within the upper 150 m via microbial respiration. Similarly, DeJong et al. [65] reported that POC export fluxes under *P. antarctica* dominance in the Ross Sea were more than twice as low as those under diatom dominance. These results suggest that a shift in the dominant phytoplankton community from diatoms to *P. antarctica* may increase microbial metabolic activity and decrease the efficiency of carbon export to the deep sea. Therefore, it is crucial to investigate the interactions among phytoplankton, microbial metabolic activity, and meltwater dynamics to better understand how shifts in phytoplankton community composition and increased meltwater inflow impact carbon cycling. Further research will provide deeper insights into the processes affecting the carbon cycle and enable a more comprehensive understanding of the factors shaping carbon dynamics in Antarctic coastal waters.

## 4. Materials and Methods

### 4.1. Study Area

The Amundsen Sea is located in West Antarctica along the Marie Byrd Land between the Bellingshausen and Ross Seas (Figure 7). The Amundsen Sea Polynya (ASP) is surrounded by Thwaites Fast ice and the Iceberg Tongue to the east, coastline including the Dotson Ice Shelf (DIS) and Getz Ice Shelf (GIS) to the south, and sea ice to the north and west [50].

### 4.2. Physico-Chemical Parameters

To examine physical and biogeochemical cycle processes, an oceanographic survey was conducted onboard the Korean icebreaker RV Araon in the Amundsen Sea during the austral summer from January to February 2016. Sample collection was divided into three regions: the ASP (P-4, 8, 10, 14, 25, 27, 29, 33), DIS (D-19, 22), and Western Getz Ice Shelf (WGIS; G-40, 42, 46, 53) (Figure 7). Water samples were collected from surface waters and subsurface chlorophyll maximum layers (SCMs) at a total of 14 stations.

The mixed layer depth (MLD) was defined as the depth at which the density difference relative to the 10 m reference depth exceeded 0.05 kg m^−3^, following the method outlined by Brainerd and Gregg [66].

Temperature and salinity were measured using a CTD device (SBE 911 Plus, Seabird Electronics, Bellevue, WA, USA). Seawater samples were collected using a Niskin bottle attached to a rosette sampler. All sample bottles were first cleaned with 10% HCl and rinsed with Milli-Q water.

Nutrient samples (50 mL) were analyzed immediately onboard the ship for dissolved inorganic nitrate (NO_3_), ammonium (NH_4_), phosphate (PO_4_), and silicate (SiO_2_) using an autoanalyzer (QuAAtro, Seal Analytical, Germany) according to the Joint Global Ocean Flux Study (JGOFS) protocols described by Gordon et al. [67].

Samples for dissolved organic carbon and nitrogen (DOC/DON) analysis were collected directly from Niskin bottles using a gravity filtering device (Holder; polycarbonate 47 mm, Advantec; GF/F filter; 0.7 µm, 47 mm, Whatman, Mumbai, India). The filtered seawater was collected into precombusted 20 mL glass ampoules (450 °C, 6 h) and stored frozen (−24 °C) until analysis. The collected DOC and DON samples were analyzed by the high-temperature catalytic oxidation method using a TOC analyzer (TOC-L, Shimadzu Corporation, Kyoto, Japan). To maintain quality, certified reference material (CRM; Batch 21 Lot#04-21, deep Florida Strait water obtained from the University of Miami, USA) was measured and validated after every six analyses.

To measure chlorophyll-a (Chl-a), 0.3 to 1 L of seawater was filtered through 47 mm GF/F, and then 90% acetone was added and extracted for 24 h in the dark at 4 °C [68]. Chl-a was analyzed immediately on board using a fluorometer (Trilogy, Turner Designs, San Joes, CA, USA). The Chl-a concentrations were then calculated from the linear relationship between the in situ fluorescence (*flu*) and fluorometer-measured concentrations of Chl-a (1):

Chl-a = 0.17 × in situ *flu* + 0.31 (r^2^ = 0.83, *p* < 0.001) (1)

### 4.3. Stable Carbon Isotope Analysis

For carbon stable isotope (δ^13^C-POC) analysis, POM samples were collected by filtering 1 L of seawater through a precombusted 25 mm GF/F filter. After removing inorganic carbon with 1 N HCl vapor, the samples were sealed in a tin capsule (8 mm × 5 mm, Isoprime, Manchester, UK) and subsequently analyzed using an elemental analyzer–isotope ratio mass spectrometer (EA–IRMS, Elemental EA–Isoprime IRMS, GV Instruments, Manchester, UK). A certified standard material (IAEA-CH-3; −24.7 ‰) was used to correct the δ (‰) of the standard gas (CO_2_ gas; 99.999%) used in the δ^13^C analysis. The analysis precision of δ^13^C was less than ±0.2‰.

Stable isotope analysis of dissolved organic carbon (δ^13^C–DOC) was performed in precombusted 40 mL amber bottles (450 °C, 6 h) after filtering with 47 mm GF/F. The collected DOC samples were adjusted to below pH 2 using 1 N HCl to remove inorganic carbon and then stored frozen until analysis [69]. The δ^13^C-DOC was determined using an isotope ratio mass spectrometer (Isoprime VisION; Elementar UK Ltd., Manchester, UK) with a TOC analyzer (Vario TOC Cube; Elementar Analysensysteme GmbH, Langensellbold, Germany). The international reference standard material Vienna PeeDee Belemnite procured from IAEA, Vienna, Austria, was used for the stable isotope analysis of δ^13^C. The δ^13^C value was standardized using IAEA-CH-6 (Sucrose International Atomic Energy Agency, −10.449‰) at an analytical precision of ±0.1‰ [69]. The δ^13^C (‰) of POC and DOC was calculated using the following Equation (2):

δ^13^C (‰) = [(R_sample_/R_standard_) − 1] × 1000 R = ^13^C/^12^C (2)

### 4.4. Hydrolysis Particulate Amino Acid

Samples for analysis of particulate amino acid (PAA) were collected by filtering 0.5–2 L of seawater through a glass fiber filter (47 mm, GF/F) precombusted at 450 °C for 6 h. For analysis of D-enantiomer (D-AA) and L-enantiomer (L-AA) amino acids in POM, the filters were freeze-dried, and 5 mL of 6 N HCl and 50 µL of 11 mM ascorbic acid were added [70]. The sample bottles were flushed with N_2_ and hydrolyzed using a microwave (Discover, CEM Corporation, Matthews, NC, USA) at 150 °C for 30 min [71]. The hydrolyzed samples were cooled at room temperature and filtered through a 0.2 μm PTFE syringe filter (Advantec, Tokyo, Japan). Each liquid phase was purged with N_2_ gas to remove HCl. The dried residue was reconstituted with 200 μL of 0.1 N HCl and transferred to a 1 mL glass vial for analysis. The D-AA and L-AA of amino acids were analyzed using an HPLC system (Agilent 1260, Agilent Technologies, Santa Clara, CA, USA) equipped with a fluorescence detector (excitation: 330 nm; emission: 450 nm). Amino acid enantiomers were separated on a Poroshell HPH-C18 column (3.0 × 150 mm, 2.7 μm, Agilent Technologies, Santa Clara, CA, USA). The automated derivatization of amino acids was performed using o-phthaldialdehyde and N-isobutyryl-l-cysteine [70]. The HPLC solvent conditions used a binary gradient. Mobile phase A used 50 mM sodium acetate (pH 6.0) buffer, and mobile phase B used acetonitrile/methanol/distilled water at 45/45/10. The conditions for amino acid analysis using the HPLC system were determined by modifying Agilent application notes [72]. The following gradient was used: 0 to 2 min, 4% B; 2 to 4 min, 10% B; 4 to 15 min, 20% B; 15 to 27 min, 35% B; 27 to 35 min, 50% B; 35 to 40 min, 100% B; 40 to 45 min, 100% B. Acid-catalyzed racemization was modified according to Kaiser and Benner [8]. The retention time and peak area precision of each AA compound were less than 0.1–0.3 and 0.5–3.0%, respectively. The concentration of PAA was determined as the sum of the concentrations of each AA. The POC normalized yield of PAA was calculated as the percentage contribution of AA carbon to the total POC concentration (3):

PAA-C (%) = [(PAA-C/POC)] × 100(3)

where POC is the bulk carbon concentration of POM, and PAA-C is the carbon number of each AA multiplied by the analysis concentration.

The degradation index (DI) of POM was calculated based on principal component analysis (PCA) and the molar ratio of amino acids as shown in Equation (4) [4]:

(4) $$DI=\sum \left[({var}_{i}-{AVG\;var}_{i})/{SD\;var}_{i}\times fac.\;coeff\right]$$

where *var_i_* is the mole percentage of AA*_i_*; *AVG var_i_* and *SD var_i_* are the mean and standard deviation of the mole percent of AA*_i_*, respectively; and *fac.coeff* is the factor coefficient in the Amundsen Sea calculated from the PCA of the AA molar ratio (Table 3).

To determine the contribution of bacterial-C to POC, the C-normalized yield of specific biomarkers of bacteria (e.g., D-Glx and D-Ala) was used in the equation below (5):

% Bacterial-C =100[biomarker]_OM_/[biomarker]_bacteria_ (5)

where [Biomarker]_OM_ and [Biomarker]_Bacteria_ are the C-normalized yields of specific biomarkers (e.g., D-Glx or D-Ala) in the POM and bacterial cells, respectively. In this study, we used the C-normalized yield of D-Glx because of the low D-Ala concentration. The C-normalized yield of D-Glx used the coefficient of 48.3 nmol mg C^−1^ as reported by Kaiser and Benner [71].

### 4.5. Statistical Analysis

Statistical analysis was performed using SPSS Statistics 18. If no normal distribution was observed, the median difference was analyzed based on a nonparametric Kruskal–Wallis test followed by a Mann–Whitney post hoc test. To determine the degradation index (DI) of POM, factor coefficients for each amino acid were derived using principal component analysis (PCA).

## Figures and Tables

**Table 1:** Sampling information, physical, and biochemical parameters of the Amundsen Sea during the austral summer of 2016.

Oceanography Setting	Station	Latitude	Longitude	Water Depth	Sampling Depth	T	S	D	MLD	NO_3_	PO_4_	Chl-a
		(s)	(w)	(m)	(m)	(°C)	(psu)	(kg m^−3^)	(m)	(µM)	(µM)	(µg L^−1^)
Amundsen Sea Polynya	P-04	−72.098	−118.884	745	0	−1.57	33.4	26.9	17	17.2	1.27	4.24
					15	−1.55	33.4	26.9		19.6	1.52	5.31
	P-08	−72.800	−116.501	627	0	−0.11	33.6	27.0	17	12.9	0.84	3.40
					20	0.11	33.8	27.2		16.5	1.06	3.86
	P-10	−73.040	−115.725	710	0	0.25	34.0	27.3	27	19.8	1.50	2.07
					20	0.24	34.0	27.3		20.3	1.52	3.56
	P-14	−73.499	−113.999	710	0	0.09	34.0	27.3	44	22.0	1.54	1.57
					20	−0.18	34.0	27.3		22.4	1.52	2.23
	P-25	−73.699	−111.597	398	0	0.71	33.9	27.2	26	5.60	0.79	9.80
					10	0.73	33.9	27.2		10.1	1.05	11.9
	P-27	−73.283	−112.166	443	0	1.01	34.0	27.2	27	10.3	0.91	3.83
					15	0.65	34.0	27.3		14.6	1.28	10.5
	P-29	−72.846	−112.481	448	0	0.52	33.9	27.2	20	16.8	1.29	3.24
					20	0.52	33.9	27.2		16.8	1.29	4.58
	P-33	−73.499	−116.499	374	0	0.02	33.5	26.9	22	9.30	0.65	1.64
					10	0.34	34.0	27.3		13.2	0.91	1.86
	Average					0.10	33.9	27.2	25	15.5	1.18	4.60
	(SD ± 1)					(0.71)	(0.22)	(0.15)	(9)	(4.91)	(0.30)	(3.25)
Dotson Ice Shelf	D-19	−74.171	−112.528	1034	0	0.30	34.0	27.3	65	17.3	1.38	2.51
					15	0.31	34.0	27.3		18.0	1.42	5.26
	D-22	−74.171	−113.328	634	0	−0.85	33.9	27.2	30	22.6	1.89	3.17
					20	−0.74	33.9	27.2		23.7	1.92	3.91
	Average					−0.25	34.0	27.3	48	20.4	1.65	3.71
	(SD ± 1)					(0.64)	(0.06)	(0.06)	(25)	(3.22)	(0.29)	(1.18)
Getz Ice Shelf	G-40	−73.077	−128.181	560	0	−1.50	33.4	26.9	23	14.9	1.18	3.85
					30	−1.32	33.6	27.0		16.1	1.42	6.15
	G-42	−74.138	−128.213	596	0	−1.17	33.3	26.8	32	12.2	0.94	0.70
					45	−1.05	33.6	27.1		19.2	1.64	4.25
	G-46	−73.940	−127.799	780	0	−1.07	33.5	26.9	25	11.8	1.01	3.83
					38	−1.26	33.6	27.0		15.3	1.29	4.65
	G-53	−73.350	−128.050	614	0	−1.41	33.2	26.7	17	11.8	1.01	3.82
					25	−1.43	33.5	26.9		14.3	1.27	5.07
	Average					−0.34	33.8	27.1	32	14.5	1.22	4.04
	(SD ± 1)					(0.86)	(0.26)	(0.18)	(17)	(2.55)	(0.24)	(1.57)

Note: T, temperature; S, salinity; D, density; MLD, mixed layer depth.

**Table 2:** Concentrations of particulate and dissolved organic matter, carbon stable isotope compositions, and indicator amino acids in the Amundsen Sea.

Oceanography Setting	Station	Sampling Depth	POC	PON	POC/ PON	δ^13^C- POC	DOC	DON	DOC/ DON	δ^13^C- DOC	PAA	PAA-C Yield	D-AA	Bacterial-C Yield	DI
		(m)	(µM)	(µM)	-	(‰)	(µM)	(µM)	-	(‰)	(µM)	(%)	(%)	(%)	-
Amundsen Sea Polynya	P-04	0	57	8	7	−25.4	47	4	11	−33.3	3.64	28.1	7.69	10.3	1.79
		15	54	7	7	−26.2	46	1	40	−23.6	3.39	27.7	4.20	10.3	2.69
	P-08	0	67	9	7	−27.3	45	2	23	−23.6	1.21	7.68	3.92	3.31	3.58
		20	62	8	7	−27.6	51	1	36	−22.6	0.83	5.65	3.25	2.11	4.33
	P-10	0	70	9	7	−26.4	45	3	13	−24.1	3.43	22.7	4.30	12.1	1.47
		20	57	8	7	−25.0	44	2	21	−24.2	4.22	34.6	4.57	15.4	−0.86
	P-14	0	56	7	7	−25.1	49	4	12	−22.8	2.52	20.2	3.36	5.24	1.46
		20	48	6	8	−24.3	46	2	19	−24.2	1.26	11.5	3.75	2.05	0.54
	P-25	0	117	8	14	−24.1	49	3	15	−25.6	7.00	28.0	2.97	11.2	0.31
		10	73	15	5	−25.1	45	1	53	−25.1	6.83	45.3	3.20	13.5	−0.53
	P-27	0	69	9	8	−26.5	38	1	32	−26.5	3.02	21.3	3.67	6.24	−0.17
		15	72	9	8	−27.1	40	1	58	−22.3	2.54	16.1	3.63	4.87	4.42
	P-29	0	75	9	8	−27.6	36	1	29	−21.0	1.68	10.9	4.62	3.10	4.43
		20	72	8	9	−26.2	36	1	34	−21.0	2.99	18.9	4.87	4.79	5.39
	P-33	0	67	11	6	−23.9	50	3	17	−	1.78	13.4	8.01	1.48	−4.96
		10	50	7	7	−24.5	49	2	25	−	1.80	17.3	8.70	1.97	−5.60
	Average		67	9	8	−25.8	45	2	27	−24.3	3.01	20.6	4.67	6.75	1.14
	(SD ± 1)		(16)	(2)	(2)	(1.26)	(5)	(1)	(14)	(3.04)	(1.81)	(10.4)	(1.81)	(4.65)	(3.17)
Dotson Ice Shelf	D-19	0	76	10	7	−26.5	45	4	13	−23.3	8.24	51.1	3.35	12.0	4.55
		15	70	11	7	−26.5	44	3	15	−21.0	9.31	62.8	3.16	16.4	4.75
	D-22	0	57	5	11	−28.7	45	3	18	−23.5	10.2	81.3	2.87	22.6	5.00
		20	45	3	15	−27.6	40	2	21	−25.7	8.41	82.7	2.57	14.1	4.34
	Average		62	7	10	−27.3	44	3	17	−23.4	9.05	69.5	2.99	16.3	4.66
	(SD ± 1)		(14)	(4)	(4)	(1.05)	(2)	(1)	(4)	(1.89)	(0.92)	(15.2)	(0.34)	(4.57)	(0.28)
Getz Ice Shelf	G-40	0	71	10	7	−25.5	46	3	18	−26.1	2.03	15.0	2.13	26.0	−5.09
		30	70	11	7	−23.1	43	3	16	−26.9	3.41	21.0	3.33	3.16	−4.19
	G-42	0	52	5	11	−24.1	48	3	15	−25.6	1.62	14.6	2.87	4.31	−4.54
		45	57	3	16	−24.3	46	1	32	−24.2	2.15	21.9	5.05	25.0	−5.91
	G-46	0	71	8	9	−25.5	45	5	8	−25.3	3.72	29.0	2.93	14.5	−4.41
		38	60	8	9	−26.1	47	4	11	−26.2	8.06	73.8	1.84	6.87	−4.76
	G-53	0	67	6	9	−24.2	45	1	63	−25.9	8.33	64.7	2.70	18.9	−4.29
		25	69	6	9	−23.1	38	1	36	−27.1	4.58	26.8	12.5	23.6	−3.74
	Average		65	7	10	−24.5	45	3	25	−25.9	4.24	33.3	4.17	15.3	−4.62
	(SD ± 1)		(7)	(3)	(3)	(1.12)	(3)	(2)	(18)	(0.93)	(2.63)	(22.8)	3.50	(9.48)	(0.66)

Note: D-AA% = (D-AA/L-AA + D-AA) × 100. D-AA analysis was performed using four standards (D-asparagine + aspartic acid (D-Asx), glutamine + glutamic acid (D-Glx), serine (D-Ser), alanine (D-Ala)). Concentrations of AA are provided in Supplementary Material Table S1.

**Table 3:** Results of principal component analysis (PCA) of the relative abundances of individual amino acids (mol%).

Amino Acid	Factor Coefficient (First Axis)	Factor Coefficient (Second Axis)	Mean	SD
Asx	0.30	0.35	2.02	0.80
Glx	−0.36	0.09	2.22	1.83
Ser	−0.21	0.56	0.54	0.42
His	−0.67	0.58	4.88	6.61
Thr	0.91	0.13	3.77	1.74
Gly	0.52	0.68	26.4	7.01
Arg	0.52	−0.08	6.24	2.87
Ala	0.12	−0.63	10.8	5.23
Tyr	−0.40	0.66	2.86	2.83
Cys	−0.70	−0.05	3.40	3.26
Val	−0.31	−0.66	7.41	4.86
IlU	0.04	−0.20	9.27	5.56
Phe	0.56	0.03	8.22	3.92
Leu	−0.42	−0.62	2.01	3.92
Lys	0.83	−0.18	4.72	3.23

## Data Availability

The original contributions presented in the study are included in the article and Supplementary Materials. Further inquiries can be directed to the corresponding author.

## Supplementary Materials

The following supporting information can be downloaded at: https://www.mdpi.com/article/10.3390/md22100476/s1, Figure S1: Disappearance nutrient ratios (NO_3_:PO_4_) in the Amundsen Sea; Table S1: Amino acid concentration of L-enantiomer (L-AA) and D-enantiomer (D-AA) in the Amundsen Sea.

## References

[1] Davis J., Kaiser K., Benner R. (2009). Amino Acid and Amino Sugar Yields and Compositions as Indicators of Dissolved Organic Matter Diagenesis. Org. Geochem..

[2] Gaye B., Lahajnar N., Harms N., Paul S.A.L., Rixen T., Emeis K.C. (2022). What Can We Learn from Amino Acids about Oceanic Organic Matter Cycling and Degradation?. Biogeosciences.

[3] Cowie G.L., Hedges J.I. (1992). Sources and Reactivities of Amino Acids in a Coastal Marine Environment. Limnol. Oceanogr..

[4] Dauwe B., Middelburg J.J., Herman P.M.J., Heip C.H.R. (1999). Linking Diagenetic Alteration of Amino Acids and Bulk Organic Matter Reactivity. Limnol. Oceanogr..

[5] Kaiser K., Benner R. (2008). Erratum: Major Bacterial Contribution to the Ocean Reservoir of Detrital Organic Carbon and Nitrogen. Limnol. Oceanogr..

[6] Tremblay L., Benner R. (2009). Organic Matter Diagenesis and Bacterial Contributions to Detrital Carbon and Nitrogen in the Amazon River System. Limnol. Oceanogr..

[7] Bourgoin L.H., Tremblay L. (2010). Bacterial Reworking of Terrigenous and Marine Organic Matter in Estuarine Water Columns and Sediments. Geochim. Cosmochim. Acta.

[8] Lehmann M.F., Carstens D., Deek A., McCarthy M., Schubert C.J., Zopfi J. (2020). Amino Acid and Amino Sugar Compositional Changes During In Vitro Degradation of Algal Organic Matter Indicate Rapid Bacterial Re-Synthesis. Geochim. Cosmochim. Acta.

[9] Fry B. (2006). Stable Isotope Ecology.

[10] Lamb A.L., Wilson G.P., Leng M.J. (2006). A Review of Coastal Palaeoclimate and Relative Sea-Level Reconstructions Using δ13C and C/N Ratios in Organic Material. Earth-Sci. Rev..

[11] Peterson B.J., Fry B. (1987). Stable Isotopes in Ecosystem Studies. Annu. Rev. Ecol. Syst..

[12] Barber A., Sirois M., Chaillou G., Gélinas Y. (2017). Stable Isotope Analysis of Dissolved Organic Carbon in Canada’s Eastern Coastal Waters. Limnol. Oceanogr..

[13] Lobbes J.M., Fitznar H.P., Kattner G. (2000). Biogeochemical Characteristics of Dissolved and Particulate Organic Matter in Russian Rivers Entering the Arctic Ocean. Geochim. Cosmochim. Acta.

[14] Ducklow H.W., Scofield O., Vernet M., Stammerjohn S., Erickson M. (2012). Multiscale Control of Bacterial Production by Phytoplankton Dynamics and Sea Ice along the Western Antarctic Peninsula: A Regional and Decadal Investigation. J. Mar. Syst..

[15] Williams C.M., Dupont A.M., Loevenich J., Post A.F., Dinasquet J., Yager P.L. (2016). Pelagic Microbial Heterotrophy in Response to a Highly Productive Bloom of *Phaeocystis antarctica* in the Amundsen Sea Polynya, Antarctica. Elem. Sci. Anthr..

[16] Fang L., Kim M. (2023). Radiocarbon Constraints on Carbon Release from the Antarctic Ice Sheet into the Amundsen Sea Embayment. J. Geophys. Res. Biogeosci..

[17] Hood E., Battin T.J., Fellman J., O’neel S., Spencer R.G.M. (2015). Storage and Release of Organic Carbon from Glaciers and Ice Sheets. Nat. Geosci..

[18] Wadham J.L., Hawkings J.R., Tarasov L., Gregoire L.J., Spencer R.G.M., Gutjahr M., Ridgwell A., Kohfeld K.E. (2019). Ice Sheets Matter for the Global Carbon Cycle. Nat. Commun..

[19] Arrigo K.R., van Dijken G., Long M. (2008). Coastal Southern Ocean: A Strong Anthropogenic CO_2_ Sink. Geophys. Res. Lett..

[20] Takahashi T., Sutherland S.C., Wanninkhof R., Sweeney C., Feely R.A., Chipman D.W., Hales B., Friederich G., Chavez F., Sabine C. (2009). Climatological Mean and Decadal Change in Surface Ocean pCO_2_, and Net Sea–Air CO_2_ Flux over the Global Oceans. Deep Sea Res. Part II Top. Stud. Oceanogr..

[21] Smith W.O., Barber D.G., Smith W.O., Barber D.G. (2007). Polynyas and Climate Change: A View to the Future. Elsevier Oceanography Series.

[22] Arrigo K.R., van Dijken G.L. (2003). Phytoplankton Dynamics within 37 Antarctic Coastal Polynya Systems. J. Geophys. Res. Ocean..

[23] Fragoso G.M., Smith W.O. (2012). Influence of Hydrography on Phytoplankton Distribution in the Amundsen and Ross Seas, Antarctica. J. Mar. Syst..

[24] Smith W.O., Shields A.R., Peloquin J.A., Catalano G., Tozzi S., Dinniman M.S., Asper V.A. (2006). Interannual Variations in Nutrients, Net Community Production, and Biogeochemical Cycles in the Ross Sea. Deep Sea Res. Part II Top. Stud. Oceanogr..

[25] Alderkamp A.C., Buma A.G.J., Van Rijssel M. (2007). The Carbohydrates of *Phaeocystis* and Their Degradation in the Microbial Food Web. Biogeochemistry.

[26] Delmont T.O., Hammar K.M., Ducklow H.W., Yager P.L., Post A.F. (2014). *Phaeocystis antarctica* Blooms Strongly Influence Bacterial Community Structures in the Amundsen Sea Polynya. Front. Microbiol..

[27] Rignot E., Mouginot J., Scheuchl B., Van Den Broeke M., Van Wessem M.J., Morlighem M. (2019). Four Decades of Antarctic Ice Sheet Mass Balance from 1979–2017. Proc. Natl. Acad. Sci. USA.

[28] Jenkins A., Dutrieux P., Jacobs S.S., McPhail S.D., Perrett J.R., Webb A.T., White D. (2010). Observations beneath Pine Island Glacier in West-Antarctica and Implications for Its Retreat. Nat. Geosci..

[29] Wåhlin A.K., Graham A.G.C., Hogan K.A., Queste B.Y., Boehme L., Larter R.D., Pettit E.C., Wellner J., Heywood K.J. (2021). Pathways and Modification of Warm Water Flowing beneath Thwaites Ice Shelf, West Antarctica. Sci. Adv..

[30] Cape M.R., Straneo F., Beaird N., Bundy R.M., Charette M.A. (2019). Nutrient Release to Oceans from Buoyancy-Driven Upwelling at Greenland Tidewater Glaciers. Nat. Geosci..

[31] Death R., Wadham J.L., Monteiro F., Le Brocq A.M., Tranter M., Ridgwell A., Dutkiewicz S., Raiswell R. (2014). Antarctic Ice Sheet Fertilises the Southern Ocean. Biogeosciences.

[32] Kim B.K., Lee J.H., Joo H.T., Song H.J., Yang E.J., Lee S.H., Lee S.H. (2016). Macromolecular Compositions of Phytoplankton in the Amundsen Sea, Antarctica. Deep Sea Res. Part II Top. Stud. Oceanogr..

[33] Kim B.K., Lee S.H., Ha S.Y., Jung J., Kim T.W., Yang E.J., Jo N., Lim Y.J., Park J., Lee S.H. (2018). Vertical Distributions of Macromolecular Composition of Particulate Organic Matter in the Water Column of the Amundsen Sea Polynya during the Summer in 2014. J. Geophys. Res. Ocean..

[34] Chen M., Jung J., Lee Y.K., Kim T.W., Hur J. (2019). Production of Tyrosine-Like Fluorescence and Labile Chromophoric Dissolved Organic Matter (DOM) and Low Surface Accumulation of Low Molecular Weight-Dominated DOM in a Productive Antarctic Sea. Mar. Chem..

[35] Son J., Jung J., Lee Y., Kim T.W., Park J., Jeon M.H., Park M.O. (2024). Contrasting Optical Properties of Dissolved Organic Matter between Oceanic Regions near the Getz and Dotson Ice Shelves in the Amundsen Sea, West Antarctica. Mar. Chem..

[36] Jeon M.H., Jung J., Park M.O., Aoki S., Kim T.W., Kim S.K. (2021). Tracing Circumpolar Deep Water and Glacial Meltwater Using Humic-Like Fluorescent Dissolved Organic Matter in the Amundsen Sea, Antarctica. Mar. Chem..

[37] Jacobs S.S., Jenkins A., Giulivi C.F., Dutrieux P. (2011). Stronger Ocean Circulation and Increased Melting under Pine Island Glacier Ice Shelf. Nat. Geosci..

[38] Randall-Goodwin E., Meredith M.P., Jenkins A., Yager P.L., Sherrell R.M., Abrahamsen E.P., Guerrero R., Yuan X., Mortlock R.A., Gavahan K. (2015). Freshwater Distributions and Water Mass Structure in the Amundsen Sea Polynya Region, Antarctica. Elem. Sci. Anthr..

[39] Mankoff K.D., Jacobs S.S., Tulaczyk S.M., Stammerjohn S.E. (2012). The Role of Pine Island Glacier Ice Shelf Basal Channels in Deep-Water Upwelling, Polynyas, and Ocean Circulation in Pine Island Bay, Antarctica. Ann. Glaciol..

[40] Lee Y., Yang E.J., Park J., Jung J., Kim T.W., Lee S.H. (2016). Physical-Biological Coupling in the Amundsen Sea, Antarctica: Influence of Physical Factors on Phytoplankton Community Structure and Biomass. Deep Sea Res. Part I Oceanogr. Res. Pap..

[41] Fang L., Lee S., Lee S.A., Hahm D., Kim G., Druffel E.R.M., Hwang J. (2020). Removal of Refractory Dissolved Organic Carbon in the Amundsen Sea, Antarctica. Sci. Rep..

[42] Lee Y., Jung J., Kim T.W., Yang E.J., Park J. (2022). Phytoplankton Growth Rates in the Amundsen Sea (Antarctica) during Summer: The Role of Light. Environ. Res..

[43] Arrigo K.R., Robinson D.H., Worthen D.L., Dunbar R.B., DiTullio G.R., VanWoert M., Lizotte M.P. (1999). Phytoplankton Community Structure and the Drawdown of Nutrients and CO_2_ in the Southern Ocean. Science.

[44] Lee Y.C., Park M.O., Jung J., Yang E.J., Lee S.H. (2016). Taxonomic Variability of Phytoplankton and Relationship with Production of CDOM in the Polynya of the Amundsen Sea, Antarctica. Deep Sea Res. Part II Top. Stud. Oceanogr..

[45] Fabiano M., Povero P., Danovaro R. (1993). Distribution and Composition of Particulate Organic Matter in the Ross Sea (Antarctica). Polar Biol..

[46] Yager P., Sherrell R., Stammerjohn S., Ducklow H., Schofield O., Ingall E., Wilson S., Lowry K., Williams C., Riemann L. (2016). A Carbon Budget for the Amundsen Sea Polynya, Antarctica: Estimating Net Community Production and Export in a Highly Productive Polar Ecosystem. Elem. Sci. Anthr..

[47] Keil R.G., Mayer L.M., Quay P.D., Richey J.E., Hedges J.I. (1997). Loss of Organic Matter from Riverine Particles in Deltas. Geochim. Cosmochim. Acta.

[48] Megens L., Van Der Plicht J., De Leeuw J.W., Smedes F. (2002). Stable Carbon and Radiocarbon Isotope Compositions of Particle Size Fractions to Determine Origins of Sedimentary Organic Matter in an Estuary. Org. Geochem..

[49] Schouten S., Klein Breteler W.C.M., Blokker P., Schogt N., Rijpstra W.I.C., Grice K., Baas M., Sinninghe Damsté J.S. (1998). Biosynthetic Effects on the Stable Carbon Isotopic Compositions of Algal Lipids: Implications for Deciphering the Carbon Isotopic Biomarker Record. Geochim. Cosmochim. Acta.

[50] Laane R.W.P.M., Turkstra E., Mook W.G. (1990). Stable Carbon Isotope Composition of Pelagic and Benthic Organic Matter in the North Sea and Adjacent Estuaries. Facets of Modern Biogeochemistry.

[51] Benner R., Fogel M., Sprague E., Hodson R. (1987). Depletion of ^13^C in Lignin and Its Implications for Stable Carbon Isotope Studies. Nature.

[52] Van Dongen B.E., Schouten S., Sinninghe Damsté J.S. (2002). Carbon Isotope Variability in Monosaccharides and Lipids of Aquatic Algae and Terrestrial Plants. Mar. Ecol. Prog. Ser..

[53] Guo J., Achterberg E.P., Shen Y., Yuan H., Song J., Liu J., Li X., Duan L. (2023). Stable Carbon Isotopic Composition of Amino Sugars in Heterotrophic Bacteria and Phytoplankton: Implications for Assessment of Marine Organic Matter Degradation. Limnol. Oceanogr..

[54] Jo N., La H.S., Kim J.H., Kim K., Kim B.K., Kim M.J., Son W., Lee S.H. (2021). Different Biochemical Compositions of Particulate Organic Matter Driven by Major Phytoplankton Communities in the Northwestern Ross Sea. Front. Microbiol..

[55] Hubberten U., Lara R.J., Kattner G. (1995). Refractory Organic Compounds in Polar Waters: Relationship between Humic Substances and Amino Acids in the Arctic and Antarctic. J. Mar. Res..

[56] Tremblay L., Caparros J., Leblanc K., Obernosterer I. (2015). Origin and Fate of Particulate and Dissolved Organic Matter in a Naturally Iron-Fertilized Region of the Southern Ocean. Biogeosciences.

[57] Kaiser K., Benner R. (2009). Biochemical Composition and Size Distribution of Organic Matter at the Pacific and Atlantic Time-Series Stations. Mar. Chem..

[58] Wei W., Blankenship D.D., Greenbaum J.S., Gourmelen N., Dow C.F., Richter T.G., Greene C.A., Young D.A., Lee S.H., Kim T.W. (2020). Getz Ice Shelf Melt Enhanced by Freshwater Discharge from beneath the West Antarctic Ice Sheet. Cryosphere.

[59] Brogi S.R., Ha S.Y., Kim K., Derrien M., Lee Y.K., Hur J. (2018). Optical and Molecular Characterization of Dissolved Organic Matter (DOM) in the Arctic Ice Core and the Underlying Seawater (Cambridge Bay, Canada): Implication for Increased Autochthonous DOM during Ice Melting. Sci. Total Environ..

[60] Thomas D.N., Lara R.J., Eicken H., Kattner G., Skoog A. (1995). Dissolved Organic Matter in Arctic Multi-Year Sea Ice during Winter: Major Components and Relationship to Ice Characteristics. Polar Biol..

[61] Deppeler S.L., Davidson A.T. (2017). Southern Ocean Phytoplankton in a Changing Climate. Front. Mar. Sci..

[62] Le Quéré C., Rödenbeck C., Buitenhuis E.T., Conway T.J., Langenfelds R., Gomez A., Labuschagne C., Ramonet M., Nakazawa T., Metzl N. (2007). Saturation of the Southern Ocean CO_2_ Sink due to Recent Climate Change. Science.

[63] Nissen C., Vogt M. (2021). Factors Controlling the Competition between *Phaeocystis* and Diatoms in the Southern Ocean and Implications for Carbon Export Fluxes. Biogeosciences.

[64] Ducklow H.W., Wilson S.E., Post A.F., Stammerjohn S.E., Erickson M., Lee S.H., Lowry K.E., Sherrell R.M., Yager P.L. (2015). Particle Flux on the Continental Shelf in the Amundsen Sea Polynya and Western Antarctic Peninsula. Elem. Sci. Anthr..

[65] DeJong H.B., Dunbar R.B., Koweek D.A., Mucciarone D.A., Bercovici S.K., Hansell D.A. (2017). Net Community Production and Carbon Export during the Late Summer in the Ross Sea, Antarctica. Glob. Biogeochem. Cycles.

[66] Brainerd K.E., Gregg M.C. (1995). Surface Mixed and Mixing Layer Depths. Deep Sea Res. Part I Oceanogr. Res. Pap..

[67] Gordon L.I., Jennings J.C., Ross A.A., Krest J.M. (1993). A Suggested Protocol for Continuous Flow Automated Analysis of Seawater Nutrients (Phosphate, Nitrate, Nitrite, and Silicic Acid) in the WOCE Hydrographic Program Office. Methods Man. WHPO.

[68] Parsons T.R., Maita Y., Lalli C.M. (1984). A Manual of Chemical and Biological Methods for Seawater Analysis.

[69] Kim M.S., Lee W.S., Suresh Kumar K., Shin K.H., Robarge W., Kim M., Lee S.R. (2016). Effects of HCl Pretreatment, Drying, and Storage on the Stable Isotope Ratios of Soil and Sediment Samples. Rapid Commun. Mass Spectrom..

[70] Fitznar H.P., Lobbes J.M., Kattner G. (1999). Determination of Enantiomeric Amino Acids with High-Performance Liquid Chromatography and Pre-Column Derivatisation with O-Phthaldialdehyde and N-Isobutyrylcysteine in Seawater and Fossil Samples (Mollusks). J. Chromatogr. A.

[71] Kaiser K., Benner R. (2005). Hydrolysis-Induced Racemization of Amino Acids. Limnol. Oceanogr. Methods.

[72] Kumagai H. (2017). Automated Precolumn Derivatization for the Enantioseparation of Amino Acids Using the Agilent 1290 Infinity II LC.

