# Supplementary material for: Characterization of Phytoplankton-Derived Amino Acids and Tracing the Source of Organic Carbon Using Stable Isotopes in the Amundsen Sea
Source: Mar Drugs. 2024 Oct 18;22(10):476. doi: 10.3390/md22100476 (PMC11509804; doi:10.3390/md22100476)

Supplementary material

Characterization of Phytoplankton-Derived Amino Acids and Tracing the Source of Organic Carbon Using Stable Isotopes in the Amundsen Sea

Jun-Oh Min[1], Min-Seob Kim[2], Boyeon Lee[1], Jong-Ku Gal[3], Jinyoung Jung[1], Tae-Wan Kim[1], Jisoo Park[1], Sun-Yong Ha[1]*

[1]Division of Ocean and Atmosphere Sciences, Korea Polar Research Institute (KOPRI), Incheon 21990, Republic of Korea

[2]Environment Measurement and Analysis Center, National Institute of Environmental Research (NIER), Incheon 22689, Republic of Korea

[3]East Sea Fisheries Research Institute, Environment and Fisheries Resources Research Division, National Institute of Fisheries Science (NIFS), Gangneung-si 25435, Gangwon-do, Republic of Korea

*Correspondence: Sun-Yong Ha (syha@kopri.re.kr)

Table S1. Amino acid concentration of L-enantiomer (L-AA) and D-enantiomer (D-AA) in the Amundsen Sea.

Oceanography setting	Station	Depth (m)	L-Asx (μM)	L-Glx (μM)	L-Ser (μM)	L-His (μM)	L-Thr (μM)	Gly (μM)	L-Arg (μM)	L-Ala (μM)	L-Tyr (μM)	L-Cys (μM)	L-Val (μM)	L-Ile (μM)	L-Phe (μM)	L-Leu (μM)	L-Lys (μM)	D-Asx (μM)	D-Glx (μM)	D-Ser (μM)	D-Ala (μM)	D-Phe (μM)
Amundsen Sea Polynya	P-4	0	0.05	0.08	0.01	0.00	0.16	0.98	0.18	0.59	0.05	0.00	0.47	0.14	0.33	0.07	0.25	0.03	0.21	0.04	0.01	0.03
		15	0.05	0.08	0.01	0.00	0.15	0.86	0.25	0.57	0.05	0.01	0.46	0.16	0.31	0.00	0.28	0.03	0.11	0.04	0.02	0.03
	P-8	0	0.02	0.03	0.00	0.05	0.08	0.59	0.03	0.00	0.04	0.01	0.03	0.08	0.14	0.00	0.06	0.01	0.05	0.03	0.03	0.03
		20	0.02	0.02	0.01	0.00	0.04	0.27	0.06	0.15	0.00	0.01	0.00	0.08	0.08	0.00	0.06	0.01	0.04	0.02	0.02	0.02
	P-10	0	0.07	0.10	0.01	0.04	0.15	0.72	0.23	0.54	0.06	0.12	0.43	0.23	0.28	0.00	0.30	0.03	0.02	0.00	0.00	0.01
		20	0.08	0.12	0.01	0.00	0.16	0.84	0.24	0.58	0.07	0.43	0.48	0.27	0.31	0.09	0.34	0.04	0.03	0.02	0.01	0.02
	P-14	0	0.03	0.04	0.01	0.05	0.10	0.66	0.18	0.38	0.06	0.00	0.30	0.27	0.18	0.01	0.15	0.02	0.07	0.04	0.02	0.03
		20	0.01	0.02	0.00	0.03	0.06	0.36	0.10	0.18	0.02	0.01	0.17	0.10	0.10	0.04	0.02	0.01	0.08	0.04	0.02	0.03
	P-25	0	0.09	0.09	0.01	0.11	0.28	1.46	0.46	1.06	0.06	0.04	0.73	1.29	0.61	0.31	0.18	0.04	0.05	0.03	0.01	0.02
		10	0.10	0.11	0.02	0.10	0.29	1.24	0.17	0.99	0.03	0.05	0.74	1.63	0.61	0.36	0.19	0.04	0.02	0.02	0.00	0.01
	P-27	0	0.07	0.05	0.01	0.03	0.12	0.57	0.10	0.35	0.03	0.13	0.30	0.65	0.24	0.12	0.14	0.02	0.02	0.01	0.00	0.01
		15	0.07	0.04	0.03	0.07	0.15	0.89	0.16	0.12	0.05	0.02	0.09	0.27	0.33	0.00	0.18	0.02	0.05	0.04	0.01	0.03
	P-29	0	0.05	0.02	0.02	0.03	0.11	0.46	0.09	0.08	0.04	0.03	0.07	0.21	0.22	0.00	0.17	0.01	0.08	0.07	0.01	0.05
		20	0.09	0.04	0.01	0.00	0.19	0.84	0.13	0.44	0.04	0.05	0.10	0.24	0.40	0.00	0.27	0.01	0.08	0.07	0.01	0.05
	P-33	0	0.01	0.01	0.01	0.05	0.03	0.28	0.08	0.10	0.01	0.09	0.19	0.41	0.10	0.24	0.04	0.03	0.09	0.07	0.02	0.05
		10	0.01	0.02	0.02	0.11	0.03	0.36	0.10	0.19	0.03	0.20	0.16	0.05	0.12	0.22	0.04	0.02	0.07	0.06	0.01	0.03
		Average	0.05	0.05	0.01	0.04	0.13	0.71	0.16	0.40	0.04	0.08	0.29	0.38	0.27	0.09	0.17	0.02	0.07	0.04	0.01	0.03
		(SD±1)	0.03	0.04	0.01	0.04	0.08	0.34	0.10	0.32	0.02	0.11	0.24	0.45	0.16	0.12	0.10	0.01	0.05	0.02	0.01	0.01
Dotson Ice Shelf	D-19	0	0.18	0.10	0.03	0.15	0.46	3.01	0.88	0.46	0.30	0.10	0.24	0.77	0.90	0.00	0.60	0.09	0.10	0.05	0.04	0.07
		15	0.20	0.13	0.04	0.18	0.49	3.19	1.34	0.55	0.28	0.12	0.25	0.85	0.96	0.00	0.65	0.10	0.11	0.06	0.03	0.08
	D-22	0	0.31	0.18	0.05	0.00	0.56	2.72	1.20	1.64	0.21	0.15	0.26	0.93	0.90	0.00	0.84	0.11	0.09	0.09	0.01	0.00
		20	0.17	0.11	0.03	0.00	0.40	2.67	1.04	1.13	0.26	0.09	0.20	0.80	0.76	0.00	0.54	0.08	0.06	0.07	0.01	0.00
		Average	0.21	0.13	0.04	0.08	0.48	2.90	1.11	0.94	0.26	0.12	0.24	0.84	0.88	0.00	0.66	0.09	0.09	0.07	0.02	0.04
		(SD±1)	0.07	0.04	0.01	0.10	0.07	0.25	0.20	0.55	0.04	0.03	0.03	0.07	0.09	0.00	0.13	0.01	0.02	0.02	0.02	0.04
Getz Ice Shelf	G-40	0	0.05	0.21	0.03	0.42	0.06	0.70	0.11	0.18	0.09	0.05	0.11	0.33	0.00	0.00	0.04	0.02	0.13	0.03	0.01	0.01
		30	0.10	0.03	0.05	0.69	0.10	1.19	0.10	0.32	0.18	0.12	0.32	0.13	0.00	0.00	0.06	0.04	0.03	0.02	0.04	0.02
	G-42	0	0.05	0.03	0.02	0.35	0.05	0.51	0.10	0.15	0.11	0.09	0.10	0.09	0.00	0.00	0.02	0.01	0.02	0.04	0.01	0.01
		45	0.03	0.20	0.01	0.15	0.06	0.61	0.10	0.11	0.19	0.26	0.16	0.26	0.21	0.00	0.00	0.03	0.20	0.02	0.01	0.00
	G-46	0	0.06	0.12	0.00	0.35	0.03	1.11	0.21	0.39	0.41	0.34	0.05	0.33	0.49	0.00	0.00	0.04	0.12	0.02	0.01	0.01
		38	0.16	0.06	0.09	1.31	0.13	1.81	0.38	0.74	0.96	0.40	0.22	0.93	0.88	0.00	0.00	0.04	0.11	0.01	0.04	0.00
	G-53	0	0.27	0.15	0.01	1.61	0.09	1.73	0.45	0.60	0.00	0.56	1.67	0.22	1.09	0.00	0.00	0.07	0.12	0.10	0.05	0.00
		25	0.13	0.19	0.01	0.06	0.02	0.70	0.29	0.92	0.06	0.23	0.37	0.02	0.02	0.43	0.22	0.06	0.48	0.11	0.01	0.05
		Average	0.11	0.12	0.03	0.62	0.07	1.04	0.22	0.42	0.25	0.25	0.38	0.29	0.34	0.05	0.04	0.04	0.15	0.04	0.02	0.01
		(SD±1)	0.08	0.08	0.03	0.56	0.04	0.51	0.14	0.30	0.31	0.17	0.53	0.28	0.44	0.15	0.07	0.02	0.14	0.04	0.02	0.02

Figure S1. Disappearance nutrient ratios (ΔNO_3:ΔPO_4) in the Amundsen Sea. ΔNO_3:ΔPO_4 ratios show conditions where diatoms and *D. speculum* are codominant (a), where *P. antarctica* is dominant (b), and mixed phytoplankton conditions near the glacier ice shelf (c).

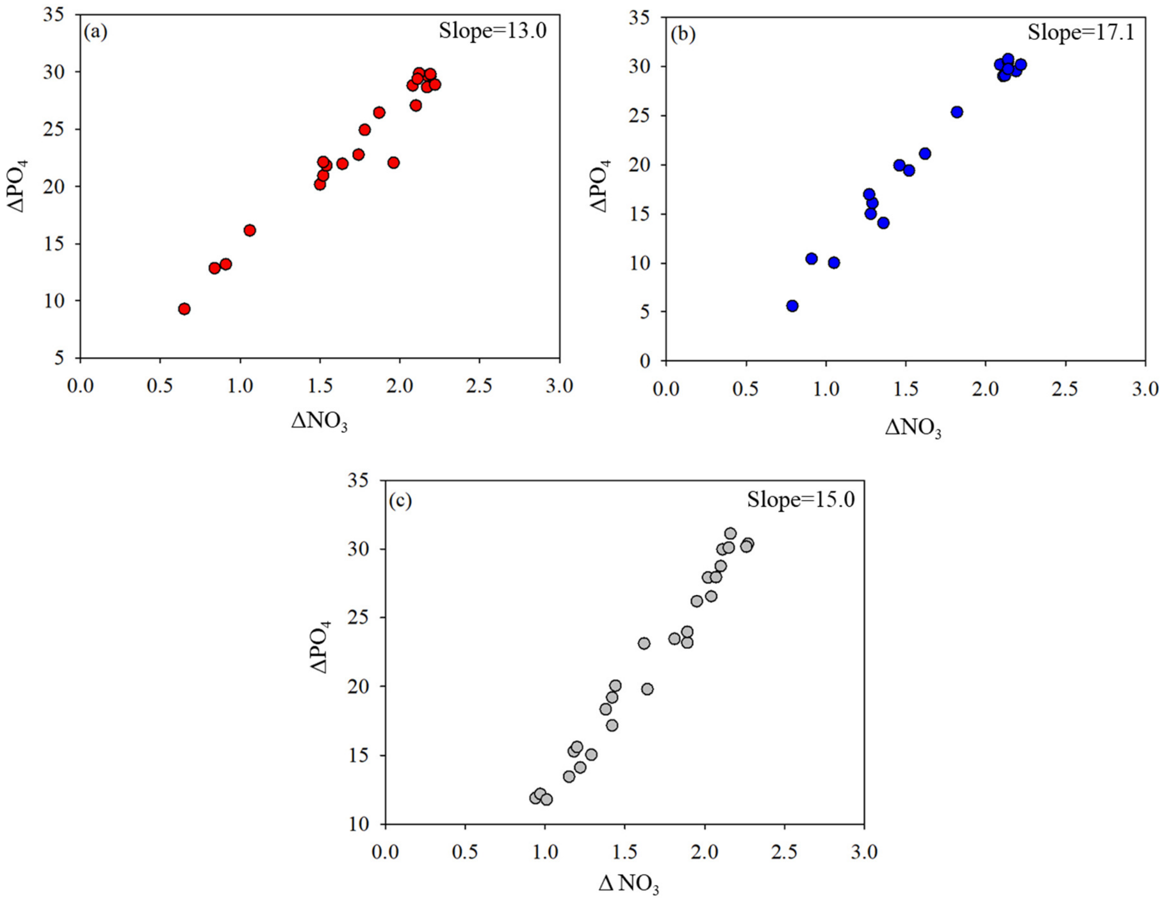

Supplement: Supplementary file 1 [file marinedrugs-22-00476-s001.zip › marinedrugs-3228308-supplementary.pdf]
